# Molecular Engineering of Electrosprayed Hydrogel Microspheres to Achieve Synergistic Anti‐Tumor Chemo‐Immunotherapy with ACEA Cargo

**DOI:** 10.1002/advs.202308051

**Published:** 2024-02-13

**Authors:** Youming Deng, Jiayang Li, Ran Tao, Ke Zhang, Rong Yang, Zhan Qu, Yu Zhang, Jinjian Huang

**Affiliations:** ^1^ Department of General Surgery Xiangya Hospital International Joint Research Center of Minimally Invasive Endoscopic Technology Equipment and Standards Central South University Changsha 410008 China; ^2^ Research Institute of General Surgery Jinling Hospital School of Medicine Nanjing University Nanjing 210002 China; ^3^ Key Laboratory of Medical Molecular Virology (MOE/NHC/CAMS) School of Basic Medical Sciences Fudan University Shanghai 200032 China

**Keywords:** ACEA, cancer, chemo‐immunotherapy, electrosprayed hydrogel microspheres, molecular design

## Abstract

Molecular engineering of drug delivering platforms to provide collaborative biological effects with loaded drugs is of great medical significance. Herein, cannabinoid receptor 1 (CB1)‐ and reactive oxygen species (ROS)‐targeting electrosprayed microspheres (MSs) are fabricated by loading with the CB1 agonist arachidonoyl 2′‐chloroethylamide (ACEA) and producing ROS in a photoresponsive manner. The synergistic anti‐tumor effects of ACEA and ROS released from the MSs are assessed. ACEA inhibits epidermal growth factor receptor signaling and altered tumor microenvironment (TME) by activating CB1 to induce tumor cell death. The MSs are composed of glycidyl methacrylate‐conjugated xanthan gum (XGMA) and Fe^3+^, which form dual molecular networks based on a Fe^3+^‐(COO^−^)_3_ network and a C═C addition reaction network. Interestingly, the Fe^3+^‐(COO^−^)_3_ network can be disassembled instantly under the conditions of lactate sodium and ultraviolet exposure, and the disassembly is accompanied by massive ROS production, which directly injures tumor cells. Meanwhile, the transition of dual networks to a single network boosts the ACEA release. Together, the activities of the ACEA and MSs promote immunogenic tumor cell death and create a tumor‐suppressive TME by increasing M1‐like tumor‐associated macrophages and CD8^+^ T cells. In summation, this study demonstrates strong prospects of improving anti‐tumor effects of drug delivering platforms through molecular design.

## Graphical abstract

Herein, arachidonoyl 2′‐chloroethylamide (ACEA)‐loaded ultraviolet‐responsive electrosprayed hydrogel microspheres (MSs) are fabricated with repeated reactive oxygen species (ROS)‐generating abilities and improved anti‐tumor functions. Specifically, ACEA can activate cannabinoid receptor 1 (CB1) and alter tumor microenvironment; concurrently, ROS produced by the MSs directly injures cancer cells, which together induces immunogenic tumor cell death. Therefore, this study shows promising prospects of improving anti‐tumor effects of drug delivering platforms through molecular engineering.

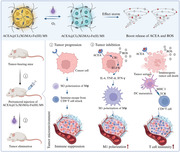

## 1 Introduction

Colorectal cancer is the third most commonly diagnosed cancer worldwide, and the second leading cause of cancer death.^[^ ^1^ ^]^ The pathogenesis of colorectal cancer involves interactions between the tumor microenvironment (TME) and tumor cells.^[^ ^2^ ^]^ Traditional treatment strategies such as surgery, chemotherapy, and radiotherapy often fail because of individual differences and tumor heterogeneity, thus allowing tumor recurrence or metastasis. Recently, immunotherapy has been recognized as a promising strategy to treat cancer. Numerous immunotherapy strategies ranging from cytokine therapy to engineered cells therapy have been developed in preclinical and clinical studies.^[^ ^3^, ^4^, ^5^ ^]^ However, limited response rate and serious adverse effects including autoimmunity and nonspecific inflammation hinder the broad implementation of these therapeutics.^[^ ^6^ ^]^ Among various factors, altered TME is responsible for cancer cell survival through immune escape.^[^ ^7^, ^8^ ^]^ Additionally, cytokine therapy and drug therapy often require intravenous injection to activate the body's immune cells to kill tumors, which can cause systemic adverse effects.^[^ ^6^ ^]^ Unfortunately, immunotherapies that can modulate TME and minimize systemic adverse effects are yet to be developed.

Cannabinoid receptors (CB), including CB1 and CB2, play important roles in various physiological processes, including memory, pain sensation, and movement.^[^ ^9^ ^]^ Mounting evidence suggests activation of cannabinoid receptors is associated with inhibition of tumorigenesis and progress in multiple tumor models.^[^ ^10^, ^11^, ^12^ ^]^ For example, CB1 activation by the selective agonist arachidonoyl 2′‐chloroethylamide (ACEA) could suppress the proliferation, migration, and invasion of colorectal cancer cells. Tumor‐associated macrophages (TAMs) are important cellular components of tumor‐infiltrating immune cells in TME.^[^ ^13^, ^14^ ^]^ M2‐like TAMs are responsible for tumor invasion, metastases, angiogenesis, and T‐cell suppression.^[^ ^15^, ^16^, ^17^ ^]^ Targeting M2‐like TAMs could alter the TME and improve tumor immunogenicity. Our previous study demonstrated that ACEA‐induced CB1 activation could suppress M2 macrophage expression in colorectal cancer by downregulating epidermal growth factor receptor (EGFR), and it could downregulate the expression of IL‐10, CCL22, Arg‐1, and CD206 in the TME.^[^ ^18^ ^]^

Notably, accumulating evidence has demonstrated that chemotherapies are promising to boost the efficacy of immunotherapy.^[^ ^19^, ^20^, ^21^ ^]^ The underlying mechanisms include the induction of tumor cell immunogenicity and the disruption of an immunosuppressive TME. ACEA, as a drug capable of altering the TME, is a promising candidate for colorectal cancer chemo‐immunotherapy. However, systemic ACEA administration may cause undesired side effects, including psychoactive effects on the central nervous system and adverse impacts on memory and mood. Because direct local injection of ACEA will lead to drug loss and uncontrolled release, the integration of ACEA with a platform capable of sustained local delivery in the tumor site may be a preferable alternative.

Electrosprayed microspheres (MSs) can act as a drug delivering platform. Electrospraying is a low‐cost, efficient, and convenient method to generate MSs from a wide range of raw materials including synthetic polymers (e.g., PLGA and PCL) and natural polymers (e.g., chitosan and alginate), driven by an appropriate electric field force.^[^ ^22^, ^23^, ^24^ ^]^ MSs have been reported to achieve sustained release of therapeutic agents in various biomedical fields such as pain relief, tissue engineering, and anti‐tumor.^[^ ^25^, ^26^, ^27^, ^28^, ^29^ ^]^ However, these MSs are usually made of inert materials such as PLGA and PCL; therefore they cannot exert biological functions on their own. The bioactivities of MSs are mainly elicited by carried drugs, which only account for a small mass proportion of the whole drug delivering platform, ranging from 4.74% to 65%.^[^ ^30^ ^]^ Granting a specific bioactivity to the MSs would supplement therapeutic functions to drug delivering platforms, but potential methods have not yet been deeply investigated.

In this study, to enhance tumor chemo‐immunotherapy, we developed a new type of electrosprayed MSs based on glycidyl methacrylate‐conjugated xanthan gum (XGMA). Once XGMA MSs were electrosprayed, the carboxyl groups of XGMA were ionically coordinated with FeCl3 solution to obtain XGMA‐Fe(III). Then, the MSs were exposed to ultraviolet (UV) light to form covalent crosslinking (CL) between C═C groups of XGMA in the presence of a photoinitiator (I‐2959). The resultant MSs were denoted as ACEA@CL(XGMA)‐Fe(III) MSs when ACEA was encapsulated. These ACEA@CL(XGMA)‐Fe(III) MSs were composed of dual networks, including the Fe^3+^‐(COO^−^)_3_ network and the C═C addition reaction network.

Notably, after soaking the ACEA@CL(XGMA)‐Fe(III) MSs in a sodium lactate buffer, the hydrogel MSs became photoresponsive because the Fe^3+^‐(COO^−^)_3_ network was disassembled by UV‐mediated reduction of Fe(III) to Fe(II), after which the MSs were renamed as ACEA @CL(XGMA)‐Fe(II) MSs. This reduction process was accompanied by consumption of sodium lactate buffer preventing aggravation of the lactate‐induced immunosuppressive microenvironment, and meanwhile promoted the massive production of reactive oxygen species (ROS), a potent anti‐tumor chemical,^[^ ^31^, ^32^, ^33^ ^]^ which contributed to killing tumor cells. Moreover, the transition of ACEA@CL(XGMA)‐Fe(III) MS's dual network to ACEA@CL(XGMA)‐Fe(II) MS's single network boosted the release of ACEA and produced synergistic anti‐tumor effects with ROS. In mouse subcutaneous colon cancer models, this dual‐targeting hydrogel MS was found to induce local inflammation and immunogenic cell death of tumor cells, which increased both M1‐like TAMs and CD8^+^ T cells, leading to reshaping of the TME and inhibition of tumor invasion. Altogether, our work demonstrated that rational molecular network design of electrosprayed hydrogel MSs may enhance tumor chemo‐immunotherapy compared to the traditional drug loading strategy.

## 2 Results and Discussion

### 2.1 Design and Regulation of Photoresponsive ACEA@CL(XGMA)‐Fe(III) MSs

**Figure 1:**
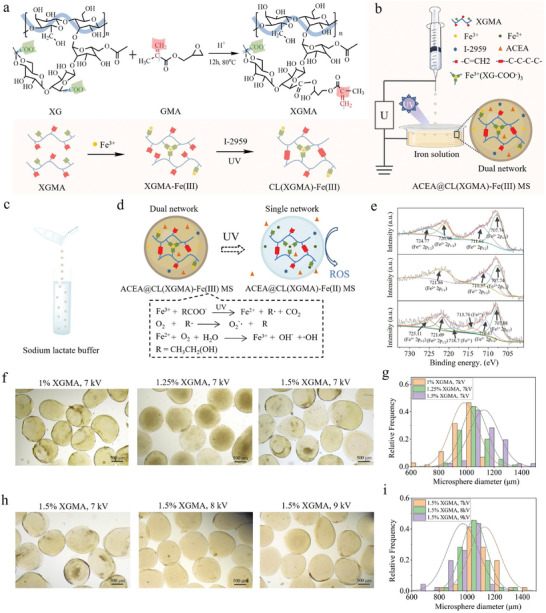
Design, fabrication, and analysis of ACEA@CL(XGMA)‐Fe(III) hydrogel MSs. a) Synthesis of XGMA by conjugating GMA to XG. b) Schematic diagram of ACEA@CL(XGMA)‐Fe(III) MS fabricated by electrospraying method (right panel). Left panel illustrates the dual‐crosslinking network within the MSs. c) Infiltrating the ACEA@CL(XGMA)‐Fe(III) MSs with sodium lactate buffer. d) UV light triggers the transformation of dual‐network MSs to single‐network MSs by reducing Fe(III) to Fe(II), which is accompanied by consumption of sodium lactate buffer and massive ROS production. e) Changes in valence state of Fe at three different fabrication stages detected by XPS. Upper panel: the stage before UV exposure; Fe(III) and Fe(II) in the hydrogel MSs were 42.89% and 57.11%, respectively. Middle panel: the stage after full UV exposure; Fe(III) and Fe(II) in the hydrogel MSs were almost 0% and 100%, respectively. Lower panel: the stage when the MSs were re‐oxidated by oxygen in air after UV exposure; Fe(III) and Fe(II) in the hydrogel MSs were 36.77% and 63.23%, respectively. The XPS data indicate that the MS can regain the ROS‐generating ability. f–i) Size regulation of ACEA@CL(XGMA)‐Fe(III) MSs. Increased XGMA concentration led to a larger diameter of the MSs (f,g), whereas increased electrospraying voltage resulted in a smaller diameter of the MSs (h,i). XG: xanthan gum; GMA: glycidyl methacrylate; XGMA: glycidyl methacrylate‐conjugated xanthan gum; CL: covalent crosslinking; MS: microsphere; ROS: reactive oxygen species; UV: ultraviolet.

The process of electrospraying MSs is dependent on electronic forces that drive the formation of small particulates from extruded liquids followed by quick solidification. In previous studies, it has been confirmed that XG solution can present a shear‐thinning property, so it is appropriate for extrusion molding.^[^ ^34^, ^35^ ^]^ On this basis, we designed the solidification strategy of XG. **Figure** **1**a indicates the conjugation of GMA to XG, thus enabling the UV curing of XG based on the addition reaction of C═C groups. The degree of substitution (DS) for GMA was calculated to be 46.07% based on the ^1^H NMR measurement (Figure S1, Supporting Information). Figure 1b demonstrates the electrospraying process of ACEA@CL(XGMA)‐Fe(III) MSs. Specifically, the modified XGMA solution with pre‐dissolved ACEA and I‐2959 was electrosprayed into FeCl_3_, which formed the first layer of the Fe^3+^‐(COO^−^)_3_ network by ionic crosslinking. Then, the MSs were exposed to UV light for the generation of a second network of ‐C‐C‐C‐C‐ groups. At this stage, the ACEA@CL(XGMA)‐Fe(III) MSs consisting of dual networks were prepared.

To endow the hydrogel MSs with photoresponsive and ROS‐generating abilities, we soaked the ACEA@CL(XGMA)‐Fe(III) MSs with a physiological reducing substance of sodium lactate (Figure 1c). In the resultant MSs, the encapsulation rate and loading capacity of ACEA were calculated to be 91.8% ± 1.3% and 3.36 × 10^−3^% ± 4.8 × 10^−5^%, respectively. Each of the five possible elements including Fe, C, O, Na, and Cl were uniformly distributed on the surface of MSs as detected by the energy dispersive spectroscopy (Figure S2, Supporting Information). This processing step could allow the ACEA@CL(XGMA)‐Fe(III) MSs to become UV sensitive since Fe(III) was reduced to Fe(II) with the assistance of sodium lactate and UV exposure. The reducing reaction was accompanied by massive production of ROS, such as superoxide anions (•O2‐) and hydroxyl radicals (OH•), which offer anti‐tumor effects (Figure 1d). At this stage of fabrication, ACEA@CL(XGMA)‐Fe(II) MSs were maintained by a single network because the Fe^3+^‐(COO^−^)_3_ network had disappeared.

To verify the reducing process, X‐ray photoelectron spectroscopy (XPS) was applied to measure the dynamic valence state change of Fe according to the Gaussian–Lorentzian curve‐fitting method.^[^ ^36^, ^37^ ^]^ The upper panel of Figure 1e shows the peaks of Fe(III) and Fe(II) in the hydrogel MSs after soaking in sodium lactate buffer, which was calculated as 42.89% and 57.11%, respectively. When fully exposed to UV light, almost 100% of the Fe in the MSs became Fe(II) (Figure 1e, middle panel). Notably, when the MSs were left in the ambient atmosphere with 21% oxygen, 36.77% of Fe(II) was re‐oxidated to Fe(III) (Figure 1e, lower panel), which reflected that the UV responsiveness of the hydrogel MSs could be recovered. Therefore, the ACEA@CL(XGMA)‐Fe(III) MSs could serve as drug carriers with endogenous anti‐tumor properties due to self‐generating ROS, and the therapeutic effects of the MSs could be reactivated due to the reversibility between Fe(II) and Fe(III).

Next, we investigated how to regulate the size of ACEA@CL(XGMA)‐Fe(III) MSs, as the size may influence the dose and suitable syringe models when locally injecting the MSs. It was found that decreasing the concentration of XGMA reduced the diameter of electrosprayed MSs (Figure 1f,g; Figure S3, Supporting Information). In addition, increasing the electrospraying voltage reduced the diameter of MSs (Figure 1h,i). All the hydrogel MSs presented a relatively uniform size under these different parameters, which suggested that electrospraying was a feasible and controllable approach for fabricating hydrogel MSs. Given that an increase in XGMA concentration could coordinate with more Fe^3+^ and the MSs in a smaller size by applying a larger voltage facilitate easy injection (Figure S4, Supporting Information), the conditions of 1.5% XGMA and 9 kV were chosen to produce the MSs used for the following experiments.

### 2.2 ACEA@CL(XGMA)‐Fe(III) MSs Boost the Production of ROS and the Release of ACEA Triggered by UV Exposure

**Figure 2:**
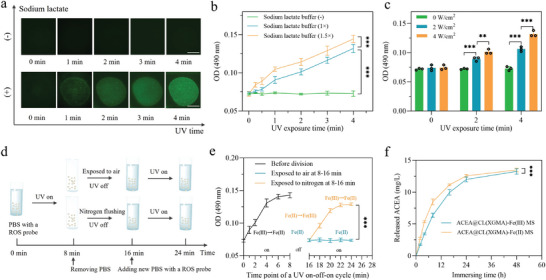
Production of ROS and boosted release of ACEA from ACEA@CL(XGMA)‐Fe(III) MS triggered by UV exposure. a) Detection of ROS production by an ROS probe in the UV‐exposed ACEA@CL(XGMA)‐Fe(III) MSs, which illustrates the necessity of pre‐treatment with sodium lactate buffer. Scale bar = 500 µm. b) Increasing the sodium lactate buffer from 1× to 1.5× accelerated ROS generation. *n* = 3. c) Increasing the UV light intensity hastened ROS production. *n* = 3. d) Experimental design for verifying whether the MSs could regain the ROS‐generating ability and exploring the potential conditions. e) Confirmation that the ROS‐generating ability was regained by the MSs in the second cycle of UV exposure when Fe(II) was re‐oxidated by oxygen in air. *n* = 3. f) Boosted release of ACEA from the MSs triggered by UV exposure due to the transformation in internal structure from dual networks to single network. *n* = 3. ^**^, *p* < 0.01; ^***^, *p* < 0.001. MS: microsphere; ROS: reactive oxygen species; UV: ultraviolet; OD: optical density; PBS: phosphate buffer solution.

Because the production of ROS by ACEA@CL(XGMA)‐Fe(III) MSs was essential to its anti‐tumor activity, we carefully studied the parameters affecting ROS production. An ROS fluorescent probe, 2,7‐dichlorodihydrouorescein diacetate (DCFH‐DA), was added to the MSs to measure ROS production during the reduction of Fe(III).^[^ ^38^, ^39^ ^]^ This probe does not show fluorescence, but instead is rapidly oxidized to a highly fluorescent molecule (2′,7′‐dichlorofluorescein) by ROS. **Figure** **2**a indicates that ACEA@CL(XGMA)‐Fe(III) MSs could boost ROS generation in the condition of sodium lactate buffer immersion and UV exposure. Without pre‐treatment with sodium lactate buffer, the MSs would not generate ROS under UV light. This implies that the use of sodium lactate is an indispensable condition for molecular engineering of ROS‐generating MSs.

The concentration of sodium lactate buffer, UV light intensity, and UV exposure time were revealed as the main influencing factors regulating the kinetics of ROS production. As shown in Figure 2b, increasing the sodium lactate buffer from 1× to 1.5× accelerated the formation and release of ROS. In addition, prolonging UV exposure time significantly promoted the accumulation of ROS. Moreover, increasing UV light intensity hastened ROS production (Figure 2c). These experimental data indicate that the generation of ROS in the ACEA@CL(XGMA)‐Fe(III) MSs can be regulated.

Because XPS detection indicated that the redox process of Fe was reversible, we wanted to explore whether this reversibility would enable ACEA@CL(XGMA)‐Fe(III) MSs to produce ROS repeatedly. To this end, we designed a parallel experiment by dividing UV‐exposed ACEA@CL(XGMA)‐Fe(II) MSs into two equal components. One was left in air to allow oxygen to re‐oxidate Fe(II) to Fe(III); the other was stored in nitrogen gas to prevent re‐oxidation of Fe(II) (Figure 2d). The results demonstrated that after the re‐oxidation of Fe(II) to Fe(III), the ACEA@CL(XGMA)‐Fe(II) MSs regained their ability to generate ROS (Figure 2e). However, without re‐oxidation, the hydrogel MSs were not able to produce ROS again when exposed to UV light. Since oxygen exists in the human body, the re‐oxidation property can allow the ACEA@CL(XGMA)‐Fe(II) MSs to produce ROS repeatedly, thereby enhancing the MSs’ anti‐tumor effects.

Because changes in the inner structures of hydrogel MSs may affect their ability to provide controlled drug release, the effects of reducing F(III) to Fe(II), which represented the transition from dual networks to a single network, on the releasing kinetics of ACEA were assessed.^[^ ^40^, ^41^ ^]^ Equal amounts of ACEA@CL(XGMA)‐Fe(III) MSs and ACEA@CL(XGMA)‐Fe(II) MSs were immersed in leach liquor under the protection of nitrogen. At the pre‐determined time points, a proportion of leach liquor was collected. High performance liquid chromatography (HPLC) was applied to measure the ACEA concentrations. The retention time of ACEA was found to be 5.547 min, and the relation of ACEA concentrations and corresponding peak area was in a very good linear correlation (R^2^ = 0.9995798), which could be used to calculate ACEA concentrations (Figure S5, Supporting Information). The results of ACEA concentrations in the leach liquor of different hydrogel MSs revealed that reducing ACEA@CL(XGMA)‐Fe(III) MSs to ACEA@CL(XGMA)‐Fe(II) MSs boosted the release of ACEA (Figure 2f). Therefore, exposing the hydrogel MSs to UV light enhances the drug delivering platform's anti‐tumor activities by increasing both ROS production and the release rate of ACEA.

### 2.3 ROS Generation and Release of ACEA from ACEA@CL(XGMA)‐Fe(III) MSs Induce Synergistic Anti‐Tumor Activity in Cancer Cells

**Figure 3:**
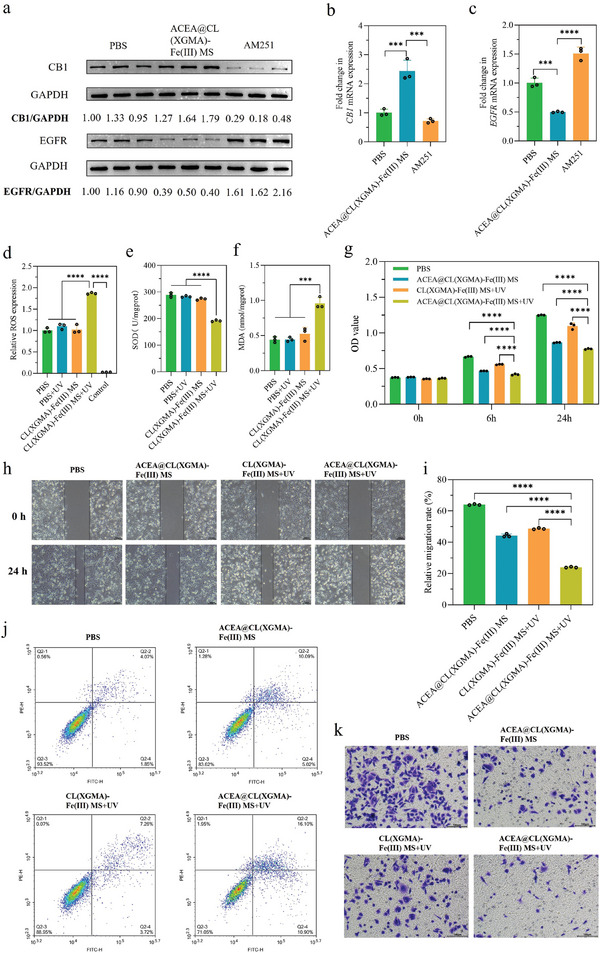
ACEA@CL(XGMA)‐Fe(III) MS + UV regulated CB1/EGFR signaling and oxidative stress in SW480 cells by releasing ACEA and generating ROS, thus offering synergistic anti‐tumor activities. a–c) ACEA released from ACEA@CL(XGMA)‐Fe(III) MS activated CB1 expression and inhibited EGFR expression (a), increased transcription of *CB1* (b), and descreased transcription of *EGFR* (c), whereas the CB1 antagonist, AM251, had opposite functions. *n* = 3. d–f) Combining CL(XGMA)‐Fe(III) MS and UV exposure significantly increased ROS production (d), lowered SOD levels (e), and improved MDA levels (f). Control: PBS‐treated SW480 cells, but without adding the ROS probe. *n* = 3. g) ACEA@CL(XGMA)‐Fe(III) MS + UV significantly inhibited proliferation of SW480 cells. *n* = 3. h) ACEA@CL(XGMA)‐Fe(III) MS + UV significantly hindered migration of SW480 cells. i) Quantitative analysis of relative migration rates in different groups. *n* = 3. j) Flow cytometry revealed the increased proportion of cell apoptosis following ACEA@CL(XGMA)‐Fe(III) MS + UV treatment. k) ACEA@CL(XGMA)‐Fe(III) MS + UV presented a synergistic inhibitory effect on invasion of SW480 cells. ^***^, *p* < 0.001; ^****^, *p* < 0.0001. MS: microsphere; CB1: cannabinoid receptor 1; EGFR: epidermal growth factor receptor; ROS: reactive oxygen species; SOD: superoxide dismutase; MDA: malondialdehyde; UV: ultraviolet; OD: optical density; PBS: phosphate buffer solution.

First, we separately verified the biological and molecular functions of the ACEA release and ROS production by ACEA@CL(XGMA)‐Fe(III) MSs and CL(XGMA)‐Fe(III) MSs + UV. **Figure** **3**a–c suggests that compared with the phosphate buffer saline (PBS) and CB1 antagonist AM251, the ACEA@CL(XGMA)‐Fe(III) MSs could activate CB1 expression and inhibit EGFR expression evidently in SW480 cells due to the release of ACEA. Then, the ability to induce oxidative stress injury of SW480 cells by CL(XGMA)‐Fe(III) MSs without loaded ACEA was examined independently. As shown in Figure 3d–f, exposing the CL(XGMA)‐Fe(III) MSs to UV light increased ROS expression, decreased superoxide dismutase (SOD) levels, and raised malondialdehyde (MDA) levels. However, separate treatments with either UV or CL(XGMA)‐Fe(III) MSs did not cause such effects. SOD is an antioxidant metalloenzyme that protects cells from oxidative stress injury, and MDA is a lipid peroxidation product. Therefore, the results indicated that combining CL(XGMA)‐Fe(III) MSs and UV exposure could generate obvious oxidative stress damages to tumor cells.

Next, we tried to determine whether the UV‐exposed ACEA@CL(XGMA)‐Fe(III) MSs, which united the ACEA's anti‐tumor function and the MSs’ ROS production ability, could generate enhanced therapeutic effects for tumors. PBS was used as a blank control, and ACEA‐loaded MSs without UV exposure or ROS‐generating MSs without loaded ACEA were used as positive controls. Figure 3g shows that UV‐exposed ACEA@CL(XGMA)‐Fe(III) MSs could inhibit the growth of SW480 cells to the greatest extent in comparison to the other control groups. Moreover, as reflected in the wound healing assay, the migration rates of the cells were the slowest in the UV‐exposed ACEA@CL(XGMA)‐Fe(III) MS group (Figure 3h,i). In addition, through detecting the cell apoptotic process by flow cytometry, it was found that the proportions of early apoptotic cells, late apoptotic cells, and total apoptotic cells were all significantly increased by treatment with UV‐exposed ACEA@CL(XGMA)‐Fe(III) MSs (Figure 3j; Figure S6, Supporting Information). Furthermore, the UV‐exposed ACEA@CL(XGMA)‐Fe(III) MSs could effectively hinder the invasion of SW480 cells compared with the other groups (Figure 3k; Figure S7, Supporting Information). Taken together, these data implied that the UV‐triggered release of ACEA from CL(XGMA)‐Fe(III) MSs and ROS regeneration synergistically promoted anti‐tumor activity against colon cancer cells, and such anti‐tumor effects were comprehensive in terms of suppressing the proliferation, migration, and invasion ability of tumor cells and promoting the apoptosis of tumor cells.

### 2.4 UV‐Responsive ACEA@CL(XGMA)‐Fe(III) MSs Exhibit Enhanced In Vivo Anti‐Tumor Activities

**Figure 4:**
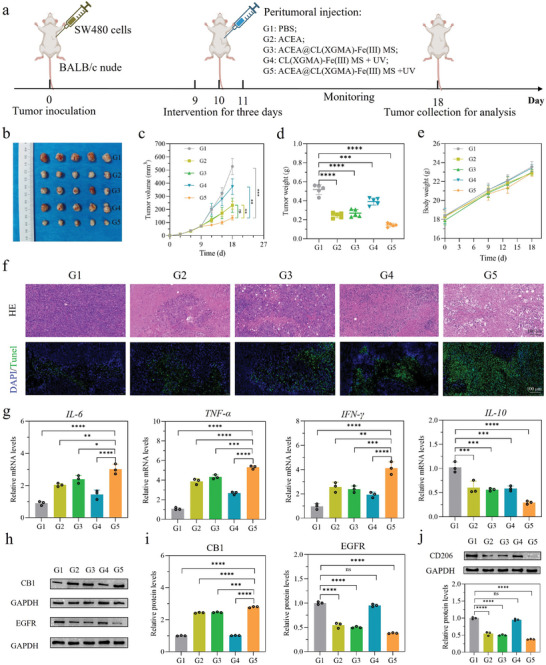
ACEA@CL(XGMA)‐Fe(III) MS + UV exhibit synergistic and enhanced in vivo anti‐tumor activities. a) Experimental process description. b) Tumor photography on day 18 in different groups. c) Comparison of tumor volume in different groups. Tumors were smallest in G5. *n* = 5. d) Comparison of tumor weight in different groups. Tumor weight was lowest in G5. *n* = 5. e) Body weight changes of tumor‐bearing mice in different groups. *n* = 5. f) HE and TUNEL staining of tumor tissues in different groups. g) Transcriptional levels of cytokines, including *IL‐6*, *TNF‐ɑ*, *IFN‐γ*, and *IL‐10*, in different groups. *n* = 3. h) Comparison of CB1 and EGFR expressions in different groups by WB. G5 presented activated CB1 and inhibited EGFR. i) Quantitative analysis of CB1 and EGFR expressions. *n* = 3. j) Detection of CD206 expression (a marker of M2 macrophages) in different groups by WB. CD206 had the lowest expression in G5. *n* = 3. ^*^, *p* < 0.05; ^**^, *p* < 0.01; ^***^, *p* < 0.001; ^****^, *p* < 0.0001; ns, not significant. G: group; MS: microsphere; UV: ultraviolet; CB1: cannabinoid receptor 1; EGFR: epidermal growth factor receptor.

Following the verification of the UV‐exposed ACEA@CL(XGMA)‐Fe(III) MSs’ enhanced anti‐tumor effects in vitro, we established a subcutaneous tumor model by injecting SW480 cells into the BALB/c nude mice to evaluate the therapeutic effects in vivo (**Figure** **4**a). Interventions by peritumor injection of different materials for each group—group 1: PBS; group 2: ACEA; group 3: ACEA@CL(XGMA)‐Fe(III) MS; group 4: CL(XGMA)‐Fe(III) MS + UV; group 5: ACEA@CL(XGMA)‐Fe(III) MS + UV—were performed on days 9–11, and the tumors were resected on day 18. The ACEA@CL(XGMA)‐Fe(III) MS was degraded more quickly and released ACEA in a faster speed in vivo following the exposure to UV when compared group 5 with group 3 (Figure S8, Supporting Information). The tumor growth inhibition effect was observed in the ACEA, ACEA@ CL(XGMA)‐Fe(III) MS, CL(XGMA)‐Fe(III) MS + UV, and ACEA@CL(XGMA)‐Fe(III) MS + UV groups based on comparison with the PBS group (Figure 4b; Figure S9, Supporting Information). Among all groups, the ACEA@CL(XGMA)‐Fe(III) MS + UV group exhibited the most retarded growth curve of average tumor volume (Figure 4c), and it presented the lowest tumor weight (Figure 4d). The body weight of mice showed no statistical differences among any groups (Figure 4e), and tissue injuries were not observed in the important organs, including heart, liver, lung, kidney, and spleen, after different treatments (Figure S10, Supporting Information). Altogether, the data suggested that the tumor inhibition ability of ACEA@CL(XGMA)‐Fe(III) MS + UV was markedly stronger than the other groups, which was attributed to the UV‐triggered accelerated release of ACEA and ROS accumulation in the tumor sites, and yet this effective treatment did not increase systemic side effects.

Then, we conducted histological analysis on the tumor tissues by H&E staining, TUNEL assay, and immunohistochemistry (IHC). H&E staining showed that there were many necrotic tumor cells in tumor tissues treated with ACEA, ACEA@ CL(XGMA)‐Fe(III) MS, CL(XGMA)‐Fe(III) MS + UV, and ACEA@CL(XGMA)‐Fe(III) MS + UV groups (Figure 4f, top panel). Among them, the ACEA@CL(XGMA)‐Fe(III) MS + UV group had the most lethal effect, whereas the PBS group caused little tumor cell death. Moreover, Ki67 IHC marking cell proliferation revealed that the tumor proliferation was inhibited to the largest extent in the ACEA@CL(XGMA)‐Fe(III) MS + UV group (Figure S11a, Supporting Information). In addition, cell death types were evaluated. The TUNEL assay revealed an evident increase of apototic tumor cells stained in green following treatment with the ACEA@CL(XGMA)‐Fe(III) MS + UV group (Figure 4f, bottom panel). GPX4 IHC and phosphorylated MLKL (p‐MLKL) IHC indicated the signicant increase in ferroptosis and necroptosis of tumor cells,^[^ ^42^, ^43^ ^]^ respectively (Figure S11b,c, Supporting Information). HMGB1, a type of damage‐associated molecular patterns (DAMPs) capable of inducing pro‐inflammatory cytokines,^[^ ^44^ ^]^ was also found to increase significantly in the ACEA@CL(XGMA)‐Fe(III) MS + UV group (Figure S11d, Supporting Information). The above evidence confirmed that UV‐exposed ACEA@CL(XGMA)‐Fe(III) MSs could suppress tumor growth by inhibiting tumor growth and inducing various types of cell death due to the synergistic effects of ACEA and ROS.

Transcription of cytokines with tumor‐regulatory activities was detected in tumor tissues by real‐time polymerase chain reaction (PCR). As shown in Figure 4g, the gene expressions of interleukin‐6 (IL‐6), interferon‐γ (IFN‐γ), and tumor necrosis factor‐α (TNF‐α) were increased in all interventional groups compared to the PBS group, and the increase in the ACEA@CL(XGMA)‐Fe(III) MS + UV group was the most significant. Moreover, the gene expression of IL‐10 was measured to be the lowest in the ACEA@CL(XGMA)‐Fe(III) MS + UV group. The differential expression of the cytokines resulted from the varying damages to tumor cells by different treatments. Moreover, the acute inflammatory responses of IL‐6, IFN‐γ, and TNF‐α have been reported to induce immunogenic cell death in tumors and promote cytotoxic adaptive immunity, whereas the anti‐inflammatory cytokine of IL‐10 has an opposite function for immune tolerance.^[^ ^45^, ^46^, ^47^ ^]^ We believe that the diverse treatments reshaped the TME differently, and the ACEA@CL(XGMA)‐Fe(III) MS + UV treatment generated acute anti‐tumor immune responses to relieve tumor progression.

We also examined whether the downstream signals of ACEA released from different hydrogel MSs were activated in vivo. Figure 4h,i reveals that direct injection of ACEA or ACEA‐loaded MSs activated CB1 and inhibited EGFR. When combined with ROS by UV exposure, the MSs further increased CB1 expression and decreased EGFR expression, suggesting synergistic effects. Moreover, CD 206 is a marker of M2‐like TAMs involved in immunosuppression of cancer.^[^ ^48^, ^49^, ^50^ ^]^ Through detecting CD 206 expression in tumor tissues via western blot (WB), it was found that the ACEA@CL(XGMA)‐Fe(III) MS + UV group showed the lowest expression of CD 206 (Figure 4j), which implied that this treatment could effectively improve the immunosuppression of cancer. Altogether, our data verify that UV‐exposed ACEA@CL(XGMA)‐Fe(III) MSs can boost the innate immunity to inhibit tumor growth by modulating cytokines and M2‐like TAMs.

### 2.5 UV‐Responsive ACEA@CL(XGMA)‐Fe(III) MSs Can Initiate Intense In Vivo Anti‐Tumor Immune Responses

**Figure 5:**
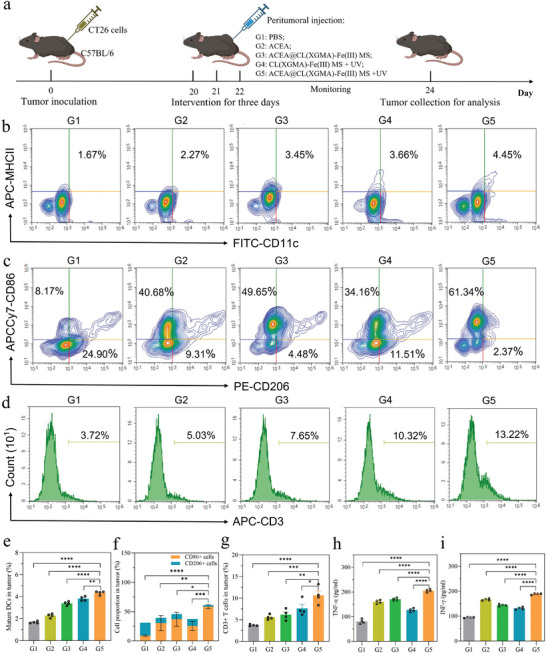
ACEA@CL(XGMA)‐Fe(III) MS + UV can initiate stronger in vivo anti‐tumor immune responses. a) Experimental process description. b) Flow cytometry revealed differential proportions of activated DCs (CD11c^+^MHCII^+^) in different groups. G5 presented the highest DC proportion. c) Flow cytometry showed differential proportions of CD86^+^ cells and CD206^+^ cells in different groups. G5 had the highest proportion of CD86^+^ cells. d) Flow cytometry indicated that the total number of T cells (CD3^+^) in tumor tissues was increased in G5. e) Quantitative analysis of DCs. *n* = 4. f) Quantitative analysis of CD86^+^ cells and CD206^+^ cells. *n* = 4. g) Quantitative analysis of T cells. *n* = 4. h) Comparison of serum TNF‐ɑ concentrations in different groups. The TNF‐ɑ concentration was highest in G5. *n* = 4. i) Comparison of serum IFN‐γ concentrations in different groups. The IFN‐γ concentration was highest in G5. *n* = 4. ^*^, *p* < 0.05; ^**^, *p* < 0.01; ^***^, *p* < 0.001; ^****^, *p* < 0.0001. G: group; MS: microsphere; UV: ultraviolet; DC: dendritic cell.

The immunosuppressive TME is responsible for tumor growth and invasion, and it has become a therapeutic target to re‐activate anti‐tumor immune responses.^[^ ^51^, ^52^, ^53^ ^]^ Therefore, we evaluated the ability of the ACEA@CL(XGMA)‐Fe(III) MS + UV treatment to re‐program the TME by measuring associated immune cells in tumor tissues dependent on flow cytometry. The animal experimental schedule is presented in **Figure** **5**a. Two days after different peritumoral treatments in CT26 tumor‐bearing female C57BL/6 mice, the tumors were harvested. Analysis of matured dendritic cells (DCs) illustrated that the proportions of matured DCs (CD11c^+^MHCII^+^) were higher in tumor tissues of mice treated with ACEA, CL(XGMA)‐Fe(III) MS + UV, or ACEA@CL(XGMA)‐Fe(III) MS + UV than those treated with PBS, and the proportion in the ACEA@CL(XGMA)‐Fe(III) MS + UV group (4.5%) was almost 2.6 times the proportion in the PBS group (Figure 5b,e). DCs have a strong antigen presentation ability and initiate immune response by capturing tumor‐associated antigens. Based on the above results, ACEA release, and ROS production could lead to the multiple types of cell death in tumors such as ferroptosis and necroptosis, and the release of DAMPs, which produced tumor‐associated antigens and increased tumor immunogenicity. Furthermore, macrophage phenotypes were explored, specifically CD86, a marker of M1 macrophages, and CD206, a marker of M2 macrophages. Reversing M2 to M1 macrophages was considered an effective treatment for reducing the immunosuppression of the TME. Through flow cytometry detection of tumor tissues, it was revealed that CD86^+^ cells in the ACEA@CL(XGMA)‐Fe(III) MS + UV group (61.34%) were significantly increased compared to other groups, whereas CD206^+^ cells were markedly decreased (2.37%) (Figure 5c,f).

To investigate whether the antigen presenting cells, including DCs and M1 macrophages, activated T cells infiltrating within the tumor tissues, CD3^+^ T cells were quantified by flow cytometry. It is shown in Figure 5d,g that the percentage of CD3^+^ T cells was significantly increased in tumor tissues treated with ACEA@CL(XGMA)‐Fe(III) MS + UV compared to PBS. CD3^+^CD8^+^ T cells, a subtype of T cells that are critical for elimination of cancer cells, were found to significantly increase in the ACEA@CL(XGMA)‐Fe(III) MS + UV group (41.37%) compared with the other groups (Figure S12, Supporting Information).^[^ ^54^, ^55^ ^]^ Of note, CD3^+^CD8^+^ T cells are usually activated by endogenous antigen presentation,^[^ ^56^, ^57^ ^]^ which reflects that the combination therapy of ACEA@CL(XGMA)‐Fe(III) MS + UV could generate more endogenous tumor antigens, resulting in an enhanced cancer cell‐killing ability. As a consequence, the serum inflammatory cytokines associated with activated T cell immunity, TNF‐ɑ, and IFN‐γ,^[^ ^58^ ^]^ were highly expressed in the ACEA@CL(XGMA)‐Fe(III) MS + UV group (Figure 5h,i), suggesting the acquisition of intensive systemic anti‐tumor immune responses. Altogether, our data verified that it is feasible to improve anti‐tumor immune responses based on rational molecular engineering of the ACEA delivering platform. Even though the proof of concept on the UV‐responsive anti‐tumor MSs has been confirmed in mouse subcutanuous tumor models, the poor penetration of UV irradition would be an issue when the MSs are translated to clinic for treatment of deep gastrointestinal tumors, which hopefully could be resolved by intergrating UV‐converting materials^[^ ^59^ ^]^ or UV‐emitting wireless devices^[^ ^60^ ^]^ into the MSs.

## 3 Conclusion

In this study, we developed UV‐responsive electrosprayed CL(XGMA)‐Fe(III) hydrogel MSs with repeated and regulatory ROS‐generating abilities based on rational molecular design of the Fe redox reaction. As a new drug delivering platform, the hydrogel MSs achieved collaborative anti‐tumor therapy with the cargo, ACEA. The proliferation, migration, survival, and invasion of cancer cells were inhibited to the largest extent after combined treatment with ACEA@CL(XGMA)‐Fe(III) MS + UV. Due to the increased immunogenic death of cancer cells, more endogenous tumor antigens were produced, which activated antigen presenting cells (e.g., DCs and M1 macrophages) and CD8^+^ T cells, causing them to generate stronger anti‐tumor immune responses and significantly inhibit tumor growth. In summary, this study offers new insights into improving the anti‐tumor activities of drug delivering platforms by not only the optimization of loaded drugs but also the reasonable molecular design of drug carriers.

## Conflict of Interest

The authors declare no conflict of interest.

## Supporting information

Supporting Information

## Data Availability

The data that support the findings of this study are available from the corresponding author upon reasonable request.
