# Supplementary material for: Molecular Engineering of Electrosprayed Hydrogel Microspheres to Achieve Synergistic Anti‐Tumor Chemo‐Immunotherapy with ACEA Cargo
Source: Adv Sci (Weinh). 2024 Feb 13;11(17):2308051. doi: 10.1002/advs.202308051 (PMC11077688; doi:10.1002/advs.202308051)
Supplement: Supplementary file 1 — Supporting Information [file ADVS-11-2308051-s001.pdf]

ADVANCED SCIENCE
Open Access

Supporting Information

for *Adv. Sci.*, DOI 10.1002/advs.202308051

Molecular Engineering of Electrosprayed Hydrogel Microspheres to Achieve Synergistic Anti-Tumor Chemo-Immunotherapy with ACEA Cargo

Youming Deng, Jiayang Li, Ran Tao, Ke Zhang, Rong Yang, Zhan Qu, Yu Zhang and Jinjian Huang**

SI appendix

Molecular Engineering of Electrosprayed Hydrogel Microspheres to Achieve Synergistic Anti-Tumor Chemo-Immunotherapy with ACEA Cargo

Youming Deng, Jiayang Li, Ran Tao, Ke Zhang, Rong Yang, Zhan Qu, Yu Zhang, Jinjian Huang

Correspondence to: Yu Zhang, e-mail: zhyu@csu.edu.cn; Jinjian Huang, e-mail: Jinjian_huang@seu.edu.cn.

This PDF file contains **Experimental methods**, and **Figures S1 to S16**.

Experimental methods

1. Materials, cell lines and animals

XG powder (MW: ~220000, viscosity of 1% aqueous solution at 20°C: 1450–2000 mPa·s) was purchased from TCI Development Co., Ltd. (Shanghai, China). GMA and 2-hydroxy-4′-(2-hydroxyethoxy)-2-methylpropiophenone (I-2959) were obtained from Aladdin Biochemical Tech. Co., Ltd. (Shanghai, China). Ferric chloride ($FeCl_3$) was purchased from Sinopharm Chemical Reagent Co., Ltd. (Shanghai, China). Sodium lactate buffer (0.5 mol/L) was prepared by mixing 1.87 mL DL-lactic acid (Sinopharm Chemical Reagent Co., Ltd., Shanghai, China) with 20 mL of 1 mol/L NaOH aqueous solution (Sigma-Aldrich, USA), followed by neutralization to pH 7 using hydrogen chloride (Sinopharm Chemical Reagent Co., Ltd., Shanghai, China). The ROS probe, DCFH-DA*,* was purchased from KeyGen Biotech Co., Ltd. (Nanjing, China). ACEA was obtained from Zzstandard® (Shanghai, China). Human colon cancer cell line SW480 and mouse colon cancer cell line CT26 were purchased from Procell (Wuhan, China) and cultured in Dulbecco’s modified Eagle medium

(DMEM) (Procell) and RPMI-1640 (Procell), respectively, which contained 10% fetal bovine serum (FBS) (Procell) and 1% penicillin-streptomycin (Procell). Female BALB/c nude mice (5 weeks old, SPF) and C57BL/6 mice (6 weeks old, SPF) were provided by Hunan Silaikejingda Experimental Animal Co., Ltd. (Changsha, China). All animal experiments were approved by the Institutional Animal Care and Use Committee of Xiangya Hospital (Approval No. 202009722) and complied with the ethical regulations.

2. Synthesis of XGMA

The conjugation of GMA to XG was based on a ring-opening polymerization reaction. Specifically, XG powder was dissolved in purified water and fully stirred to produce 0.6 wt% solution. Then, the solution's pH was adjusted to 4.2–4.8 using hydrochloric acid, followed by dropwise addition of 1% (v/v) GMA to the XG solution. The resultant solution was stirred for 12 h at 80°C. Next, the mixture was collected and dialyzed for 3 days using dialysis membranes (molecular weight cut-off: 12–14 kDa) to remove unreacted residues. The dialysate was freeze-dried for 48 h to obtain XGMA. The DS of GMA to XG was calculated based on the ratio of area at peaks of δ = 5.65 and δ = 6.08 to that of δ =1.81 by ^{1}H NMR measurement.

3. Preparation of ACEA@CL(XGMA)-Fe(III) MS

The pre-gel solution was prepared by dissolving XGMA at different concentrations of 1, 1.25, and 1.5 wt% into a mixed aqueous solution containing 0.5 wt% I-2959 and 100 μM ACEA. Then, the pre-gel solution was stored in a 10 mL syringe with a 24G metal nozzle and electrosprayed with varying voltages at 7, 8, and 9 kV at the rate of 5 mm/min by an electrospinning machine (Yongkangleye Tech Co., Ltd., Beijing, China). The electrosprayed MSs were collected in 2.4 wt% $FeCl_3$ solution, during which the first layer of Fe^{3+}-$(COO^{-})_3$ network was generated. Afterwards, the MSs were exposed to UV light to form

the second layer of C=C addition reaction network. The resultant ACEA@CL(XGMA)-Fe(III) MSs were immersed in the sodium lactate buffer for 2 min and centrifuged to harvest the MSs. The encapsulation rate of ACEA was determined by the ratio of the total amount of incorporated ACEA in the MSs to the total quantity of ACEA added initially during the preparation. The loading capacity of ACEA was calculated by the ratio of the total amount of incorporated ACEA in the MSs to the total quantity of the MSs.

4. Characterization of ACEA@CL(XGMA)-Fe(III) MS

The morphology of ACEA@CL(XGMA)-Fe(III) MSs was observed in a microscope (CKX53, Olympus, Japan), and the size of MSs was analyzed with ImageJ (NIH, USA). The elements of freeze-dried MSs were measured with the energy dispersive spectroscopy (SU8220, HITACHI, Japan). The injectability of MSs in different sizes were evaluated by injection forces measured with a universal testing machine (MTS CMT2103, USA) where the MSs were placed into a 2-mL syringe with a 16G needle and injected at the rate of 10 mm/min.

5. Detecting and explaining ROS-generating ability of the MS

To visualize ROS production in the ACEA@CL(XGMA)-Fe(III) MSs, the ROS probe was loaded in the pre-gel solution at the final concentration of 10 μM. The produced ACEA@CL(XGMA)-Fe(III) MSs loaded with the ROS probe were observed under UV exposure with a SpinSR confocal microscope (Olympus, Japan). The MSs without UV exposure were used as a control. Moreover, to investigate the parameters affecting ROS production, we adjusted the sodium lactate buffer concentration to 0 mol/L, 0.5 mol/L (1×), and 0.75 mol/L (1.5×), and the UV light intensity to 0 W/cm^2, 2 W/cm^2, and 4 W/cm^2. The MSs were dissolved in PBS containing the ROS probe at 10 μM. After different UV exposure times, ranging from 0 min to 4 min, 100 μL of PBS was collected for detection of the optical density (OD) at 490 nm using a multi-functional

microplate reader (BioTek, USA). In addition, after a cycle of UV exposure, we divided the MSs into two equal parts and placed them into air atmosphere and nitrogen environment separately for 8 min. Then, the MSs were immersed in PBS containing the ROS probe and exposed to UV light again. Subsequently, the microplate reader was used to measure the OD of PBS at 490 nm to examine whether the MSs regained their ROS-generating ability when re-oxidized by oxygen. To reveal the potential chemical mechanisms, the valence state change of Fe was evaluated based on XPS (Nexsa G2, Thermo Fisher Scientific, USA) according to the Gaussian–Lorentzian curve-fitting method.

6. Releasing profile of ACEA *in vitro*

After immersing 500 μL ACEA@CL(XGMA)-Fe(III) MSs in 1 mL PBS and exposing the mixture to UV light for 4 min followed by nitrogen protection, 50 μL of PBS was harvested at pre-determined time points of 0, 2, 4, 8, 16, 24, and 48 h. The MSs without UV exposure were used as a control. ACEA concentration in the PBS was measured using HPLC (LC20A, SHIMADZU, Japan). Water and acetonitrile were used as the mobile phase.

7. ROS generation and oxidative stress injury of SW480 cells

The ROS probe was also utilized to detect ROS levels of SW480 cells with the treatment of CL(XMGA)-Fe(III) MS + UV. Briefly, 4×10^5 cells were seeded into 12-well plates and incubated for 24 h. Then, around 100 μL of CL(XGMA)-Fe(III) MSs or PBS was added into six wells. On this basis, three of the six wells were placed under UV irradiation at 4 W/cm^2 for 1 min. After incubation for 6 h, the cells were incubated with the ROS probe. The PBS-treated cells without adding the ROS probe were used as a control. Moreover, SOD and MDA involved in the oxidative stress reaction were detected using a SOD kit (Jiancheng, Nanjing, China) and MDA kit (Solarbio, Beijing, China). The cells were processed as mentioned above. After lysis and centrifugation, the

supernatant was collected for detection of SOD and MDA.

8. WB analysis

The SW480 cells were seeded into 6-well plates and cultured for 24 h with medium supplemented with 50 μL of ACEA@CL(XGMA)-Fe(III) MSs or the CB1 antagonist AM251 (10 μM). Cell lysates were harvested by RIPA lysis buffer and then boiled with loading buffer. Moreover, collected tumor tissues were cut into pieces in culture dishes. The cells or tissues were lysed and centrifuged, and the supernatant was collected as a protein sample. Protein lysates were separated by 10% SDS-PAGE gel and then transferred onto PVDF membranes (Millipore, USA). The membranes were blocked with TBST containing 5% non-fat milk for 2 h at room temperature, followed by incubation with primary antibodies against CB1 (1:1000, 17978-1-AP, Proteintech), EGFR (1:10000, 66455-1-Ig, Proteintech; 1:10000, AF6043, Affinity), or CD206 (1:500, 18704-1-AP, Proteintech). GAPDH (1: 6000, 60004-ig-Ag, Proteintech) was used as a control band. Thereafter, the membranes were incubated with HRP-conjugated secondary antibody at room temperature for 2 h. Protein bands were visualized using a chemiluminescent imaging system (Tanon 5200 Multi, Shanghai, China).

9. Real-time PCR

Cells or tissues were lysed by TRIzol (15596026, Thermo Fisher Scientific, USA), the total RNA was isolated, and cDNA synthesis was performed with a PrimeScript RT reagent kit (K1622, Thermo Fisher Scientific, USA) according to the manufacturer's instructions. Real-time PCR was performed using a BeyoFast™ SYBR Green qPCR Mix (1708882AP, Bio-Rad, USA). All primers are listed in **Table 1**.

Table 1. Primer sequence for real-time PCR.

Gene name	Gene sequence

CB1	forward	ATGTGGACCATAGCCATTGTG
	reverse	CCGATCCAGAACATCAGGTAGG
EGFR	forward	AGGCACGAGTAACAAGCTCAC
	reverse	ATGAGGACATAACCAGCCACC
IL-6	forward	TAGTCCTTCCTACCCCAATTTCC
	reverse	TTGGTCCTTAGCCACTCCTTC
TNF-α	forward	CCCTCACACTCAGATCATCTTCT
	reverse	GCTACGACGTGGGCTACAG
INF-γ	forward	AACTCAAGTGGCATAGATGTGGAAG
	reverse	TGCTGAAGAAGGTAGTAATCAGGTG
IL-10	forward	GCTGGACAACATACTGCTAACC
	reverse	ATTTCCGATAAGGCTTGGCAA
GAPDH	forward	AGGTCGGTGTGAACGGATTTG
	reverse	TGTAGACCATGTAGTTGAGGTCA

10. CCK-8 assay

Cell proliferation was investigated using a CCK-8 kit (Servicebio, China). In short, 100 μL of cell suspensions with a density of 5 × 10^4/ml was seeded into a 96-well plate for 24 h. Then the cells were treated with 4 μL PBS, 4 μL ACEA at the concentration of 91.8 μM, 4 μL CL(XGMA)-Fe(III) MS + UV (365 nm, 4 W/cm^2, 1 min), or 4 μL ACEA@ CL(XGMA)-Fe(III) MS + UV (365 nm, 4 W/cm^2, 1 min). The cells were incubated with CCK-8 reagents at 0, 6, and 24 h. The absorbance at 450 nm was measured using the multi-functional microplate reader.

11. Wound healing test

Cell migration was investigated by the wound healing test. First, 2 × 10^5 cells in 1 mL culture medium were seeded into each well of a 24-well plate and incubated for 24 h. Next, the monolayer cells were scratched vertically with a

sterile pipette tip, and the cells were photographed with a microscope. Then, the cells were treated with 40 μL PBS, 40 μL ACEA at the concentration of 91.8 μM, 40 μL CL(XGMA)-Fe(III) MS + UV (365 nm, 4 W/cm^2, 1 min), or 40 μL ACEA@ CL(XGMA)-Fe(III) MS + UV (365 nm, 4 W/cm^2, 1 min). After being cultured for 24 h, the cells were photographed. The relative migration rate was determined by the ratio of cell movement distance to the initial gap length.

12. Transwell invasion assay

Cell invasion was determined with the transwell invasion assay. SW480 cell suspensions were prepared with FBS-free medium. Then, 100 μL of the cell suspension containing 5 × 10^4 cells was added into the upper chamber. The cells were subjected to 4 μL PBS, 4 μL ACEA at the concentration of 91.8 μM, 4 μL CL(XGMA)-Fe(III) MS + UV (365 nm, 4 W/cm^2, 1 min), or 4 μL ACEA@ CL(XGMA)-Fe(III) MS + UV (365 nm, 4 W/cm^2, 1 min) for 24 h. Subsequently, the invaded cells were stained with crystal violet and counted under a microscope.

13. Cell apoptosis analysis *in vitro*

Flow cytometry was utilized to detect cell apoptosis. Two milliliters of SW480 cell suspension containing 2× 10^6 cells was treated with 80 μL PBS, 80 μL ACEA at the concentration of 91.8 μM, 80 μL CL(XGMA)-Fe(III) MS + UV (365 nm, 4 W/cm^2, 1 min), or 80 μL ACEA@ CL(XGMA)-Fe(III) MS + UV (365 nm, 4 W/cm^2, 1 min) for 24 h. Next, 5 × 10^5 cells were collected for each group and mixed with 500 μL binding buffer to prepare cell suspensions for staining with 5 μL annexin V-FITC and 5 μL propidium iodide (KGA108-1, KeyGen Biotech Co., Ltd, Nanjing, China). After incubation for 15 min, the cells were detected using a flow cytometer (NovoCyte 2040R, ACEA, USA). The quantification of early and late apoptotic cells was carried out using FlowJo software. The gating strategy is shown in **Figure S13**.

14. *In vivo* tumor models and treatment

To evaluate the therapeutic effects of ACEA@ CL(XGMA)-Fe(III) MS + UV, 1 × 10^6 SW480 cells were injected subcutaneously into the left flank of the BALB/c nude mice. Nine days later, when the tumor volume reached ~100 mm^3, mice were randomly divided into five groups (n = 5) as follows: PBS (as control); ACEA (1.5 mg/kg/d); ACEA@CL(XGMA)-Fe(III) MS; CL(XGMA)-Fe(III) MS + UV; and ACEA@CL(XGMA)-Fe(III) MS + UV. The ACEA in the MSs was used in the same dose as that injected directly. For the two groups treated with UV, the mice were treated with 5 min of UV irradiation (365 nm, 4 W/cm^2, interval time: 6 h) 3 times per day for 3 d at the tumor sites. Tumor volume and body weight of mice were measured at 0, 3, 6, 9, 12, 15, and 18 d. Finally, the mice were sacrificed, and the tumor and major organ tissue were harvested for subsequent analysis.

To compare the profile of controlled ACEA release from MSs in the ACEA@CL(XGMA)-Fe(III) MS group and ACEA@CL(XGMA)-Fe(III) MS + UV group, from the day 9 to day 19 after the injection of MSs, the skin covered on tumors was cut. The residual MSs were taken out carefully, and then weighed followed by the placement in tubes. The amount of drug in the residual hydrogel was determined by HPLC. Water and acetonitrile were used as the mobile phase. The degradation of MSs and the accumulated release of ACEA in the two groups were calculated by the following equations:

$$\textit{In vivo}\ \text{MS degradation} = (M_{ms@0} - M_{ms@n}) / M_{ms@0} \times 100\%;$$

$$\text{Accumulated ACEA release} = (M_{drug@0} - M_{drug@n}) / M_{drug@0} \times 100\%;$$

$M_{ms@0}$ is the initial mass of MSs injected to tumor sites, and $M_{ms@n}$ is the residual mass of MSs on the day n of injection; $M_{drug@0}$ is the initial mass of ACEA in the MSs injected to tumor sites, and $M_{drug@n}$ is the residual mass of ACEA in the MSs on the day n of injection.

In addition, to study the immune responses of ACEA@ CL(XGMA)-Fe(III)

MS + UV, a different tumor model was established by subcutaneously injecting 1×10^6 CT26 cells into the left flank of the C57BL/6 mice. When the tumor volume reached ~100 mm^3 after 20 days, mice were treated peritumorally in the left flank with PBS (as control), ACEA (1.5 mg/kg/d), ACEA@ CL(XGMA)-Fe(III) MS, CL(XGMA)-Fe(III) MS + UV, or ACEA@ CL(XGMA)-Fe(III) MS + UV. The ACEA in the MSs was used in the same dose as that injected directly. For the two groups treated with UV, the mice were treated with 5 min of UV irradiation (365 nm, 4 W/cm^2, interval time: 6 h) 3 times per day for 3 d at the tumor sites. Two days later, the tumors were resected for subsequent analysis.

15. Hematoxylin & eosin (HE) staining

The tumors and organ tissues, including heart, liver, lung, kidney, and spleen, were collected, and fixed with formalin, dehydrated, and then embedded in paraffin. Serial sections were prepared, subjected to HE staining, and photographed with a microscope.

16. TUNEL staining

The tumors were fixed in formalin, embedded in paraffin, and sectioned. A TUNEL kit (TUN11684817, Roche, Swiss) was used to detect the apoptosis of the tumor tissue according to the manufacturer's instructions.

17. IHC

The paraffin sections of tumors were dewaxed to water followed by antigen repair. Then, 3% hydrogen peroxide solution was used to block endogenous peroxidase, and 3% bovine serum albumin solution as a blocking reagent was applied for incubation. Different primary antibodies including Ki67 (1:300, GB111499, Servicebio), GPX4(1:200, GB114327, Servicebio), p-MLKL (1:200, ab196436, Abcam), and HMGB1 (1:1500, GB11103, Servicebio) were incubated with sliced tissues at 4°C overnight, and then treated with secondary

antibodies. After staining with DAB color developing solution (G1212, Servicebio) and hematoxylin labeling nuclei, the slides were observed under the microscopy and positive signals were calculated with ImageJ.

18. Flow cytometry *in vivo*

The tumors harvested from mice were divided into pieces, and cell suspensions were prepared in the presence of digestive enzymes. The cells were stained with fluorescence-labeled antibodies including CD11c (1:200, 11-0114-82, Invitrogen), MHCII (1:200, 47-5321-82, Invitrogen), CD86 (1:200, 47-0862-82, Invitrogen), CD206 (1:200, 12-2061-82, Invitrogen), CD3 (1:200, 47-0032-82, Invitrogen), CD4 (1:200, 45-0049-42, Invitrogen), and CD8 (1:200, MHCD0801, Invitrogen) following the manufacturer's instructions. The stained cells were detected with a flow cytometer (MateCyte2L6C, Challenbio, Beijing, China). The data were analyzed with FlowJo software. The gating strategy of activated DCs ($CD11c^+MHCII^+$) is shown in **Figure S14**. The gating strategy of $CD86^+$ cells and $CD206^+$ cells is shown in **Figure S15**. The gating strategy of T cells ($CD3^+$) and the subtypes of $CD4^+$ T cells and $CD8^+$ T cells is shown in **Figure S16**.

19. ELISA assay

Serum samples were isolated from C57BL/6 mice. Then, the levels of TNF-α (RX202412M, Ruixin Biotech, Quanzhou, China) and INF-γ (RX203097M, Ruixin Biotech, Quanzhou, China) were measured with the corresponding ELISA kits according to the manufacturer’s instructions.

20. Statistical analysis

Statistical analysis was performed using Origin Pro and GraphPad Software 9.0. All data were presented as the mean value ± standard error of the mean (SEM), and analyzed with one-way ANOVA (when there were more

than two groups) or two-way ANOVA (when there were two influencing factors). Tukey's multiple comparisons test was performed when necessary. $P < 0.05$ was considered to indicate statistical significance.

Supplementary Figures

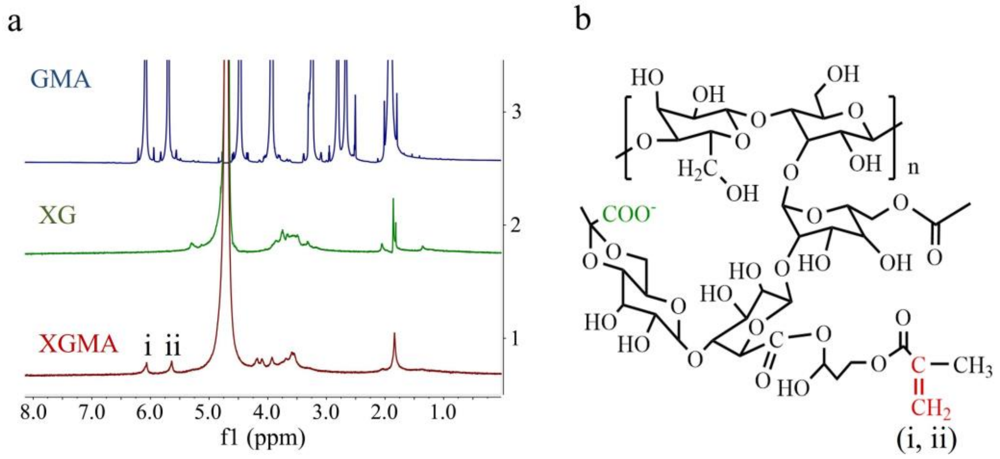

Figure S1, related to Figure 1a. Confirmation on the successful synthesis of XGMA. (a, b) ^{1}H NMR measurement of GMA, XG, and XGMA (a), in which the signals at δ = 6.07 (i) and 5.66 (ii) referred to the vinyl protons (b), suggesting the presence of GMA on XG. XG: xanthan gum; GMA: glycidyl methacrylate; XGMA: glycidyl methacrylate-conjugated xanthan gum.

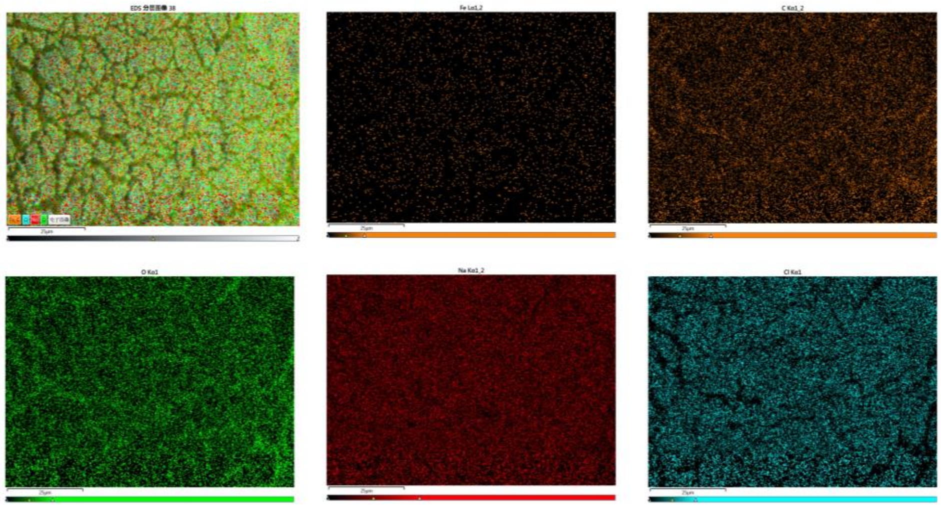

Figure S2, related to Figure 1c. Analysis of elements in ACEA@CL(XGMA)-Fe(III) MS using an energy dispersive spectrometer. Five elements (Fe, C, O, Na, and Cl) were uniformly distributed on the surface of MS. MS: microsphere.

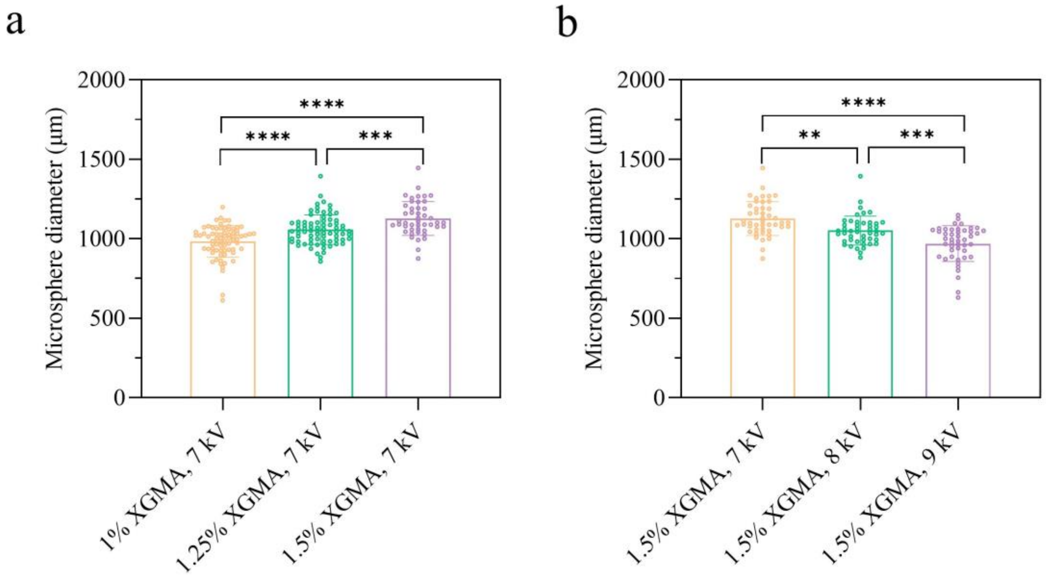

Figure S3, related to Figure 1g and i. (a) Increase in XGMA concentration increases the diameter of microspheres. n = 73 (left column), 75 (middle column), and 46 (right column). (b) Increase in voltage decreases the diameter of microspheres. n = 46. **, $P < 0.01$; ***, $P < 0.001$; ****, $P < 0.0001$. XGMA: glycidyl methacrylate-conjugated xanthan gum.

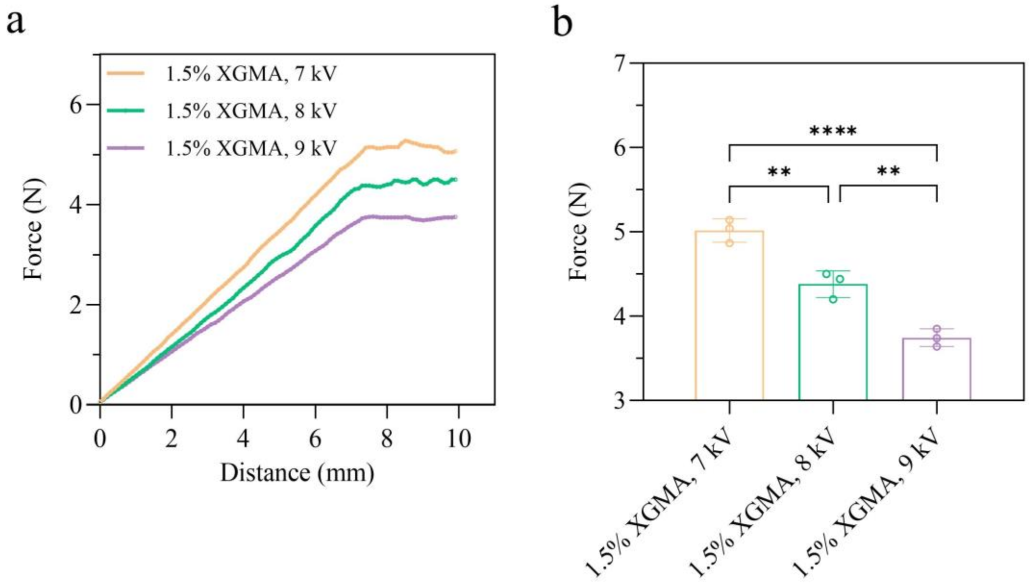

Figure S4. MSs in a smaller size by applying a larger voltage facilitate easy injection. (a) Force-distance curves for the injection of different MSs. (b) Quantitative analysis of injection force for different MSs. **, $P < 0.01$; ****, $P < 0.0001$. MS: microsphere; XGMA: glycidyl methacrylate-conjugated xanthan gum.

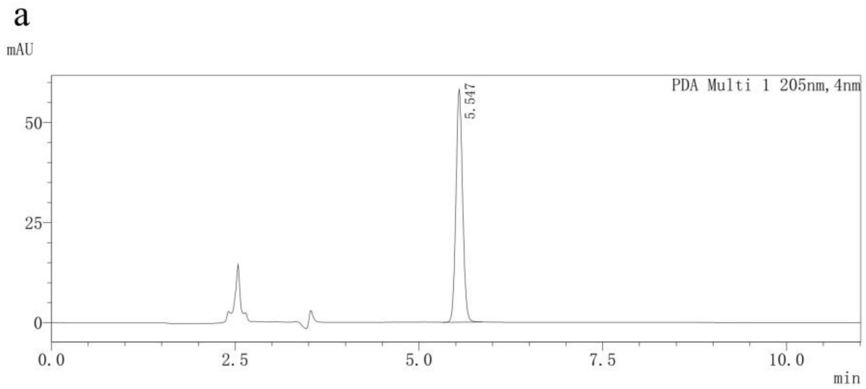

b

#	Concentration (mg/L)	Area
1	1.5	20109
2	3	38694
3	6	67913
4	15	182853
5	30	365633

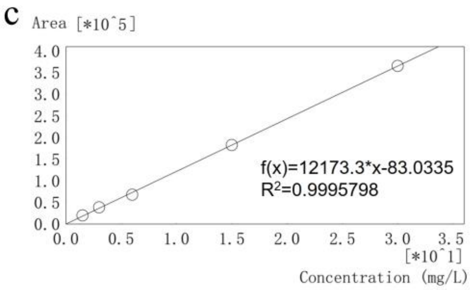

Figure S5, related to Figure 2f. Detection of ACEA concentration using HPLC. (a) Determination of ACEA retention time as 5.547 min. (b, c) Preparation of ACEA concentration gradients (b) to establish of a standard concentration-area curve (c). HPLC: high performance liquid chromatography.

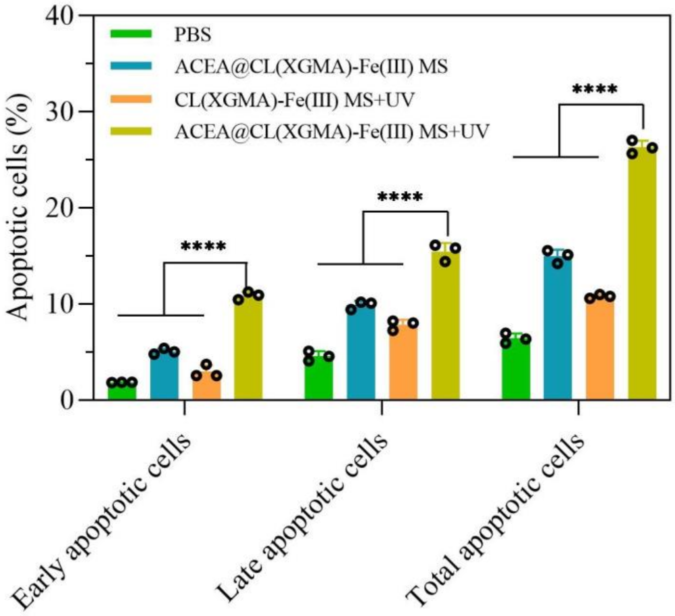

Figure S6, related Figure 3j. Quantitative analysis of early and late apoptotic cell proportions after different treatments based on the outcome of flow cytometry. n = 3. ****, $P < 0.0001$.

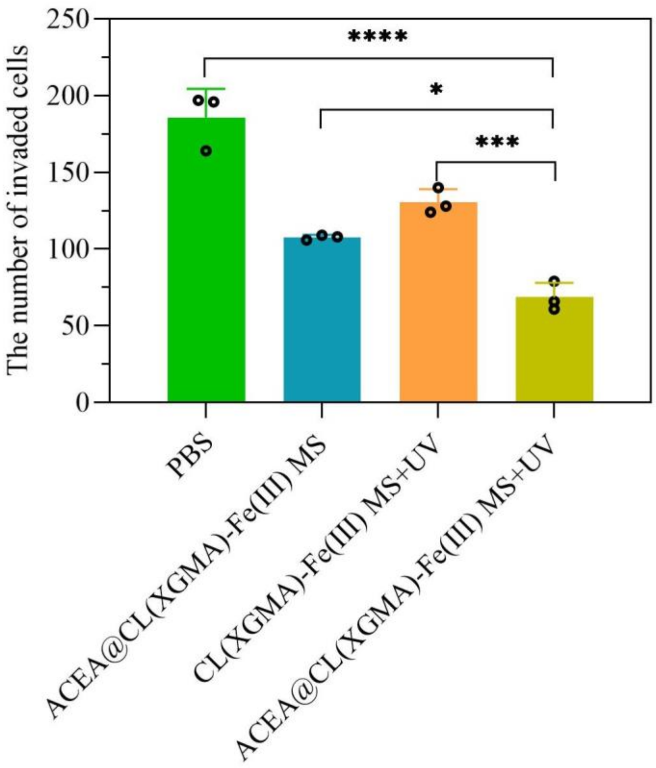

Figure S7, related to Figure 3k. Quantitative analysis of invaded cell counts after different treatments based on the Transwell invasion assay. n = 3. *, $P < 0.05$; ***, $P < 0.001$; ****, $P < 0.0001$. PBS: phosphate buffer solution; MS: microsphere; UV: ultraviolet.

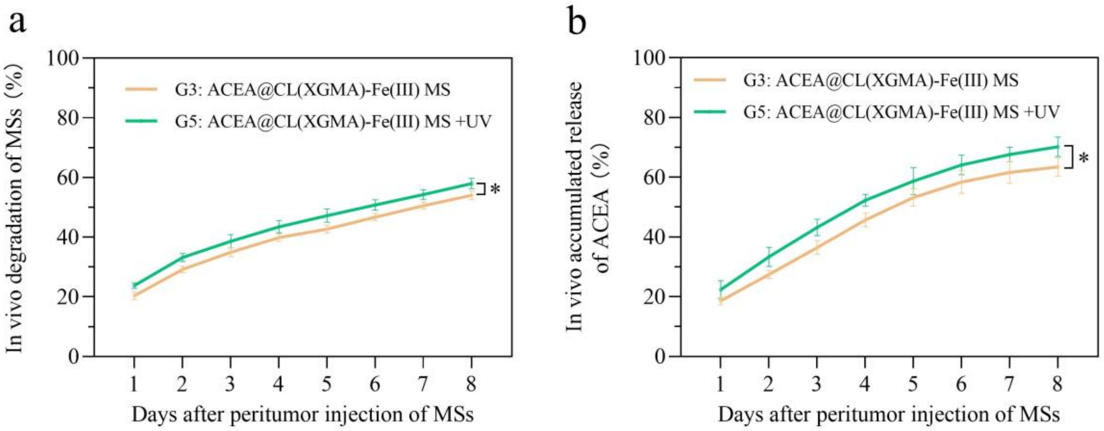

Figure S8. The *in vivo* MS degradation (a), and ACEA releasing patterns from the MSs (b) with or without UV exposure. n = 3. *, $P < 0.05$. MS: microsphere; UV: ultraviolet.

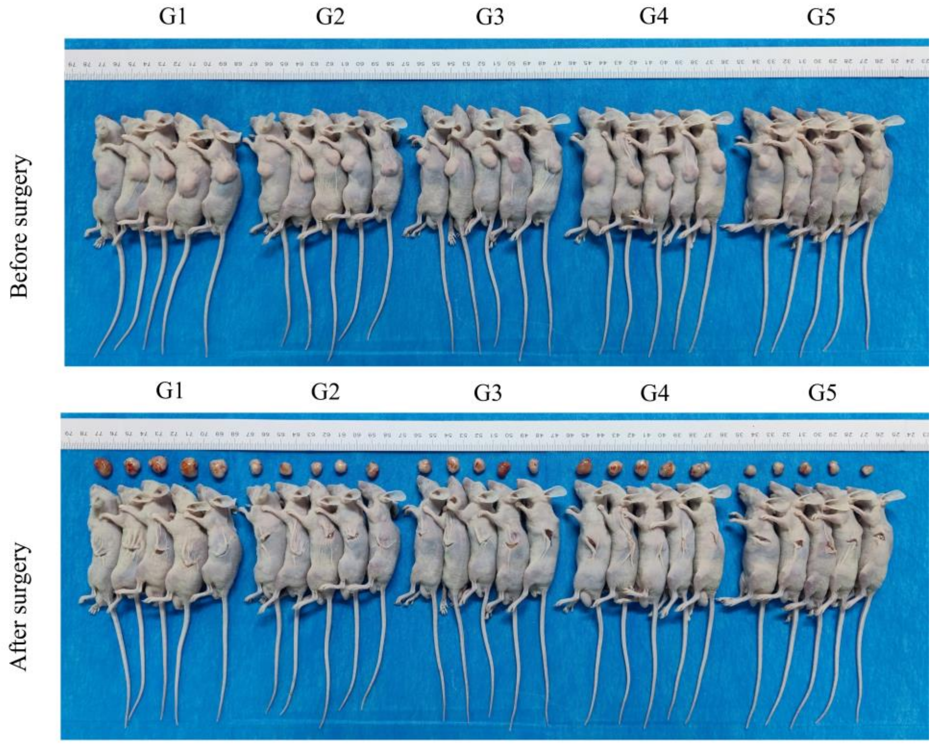

Figure S9, related to Figure 4b. Photography of tumors in different groups before (upper panel) and after (lower panel) surgery. G: group.

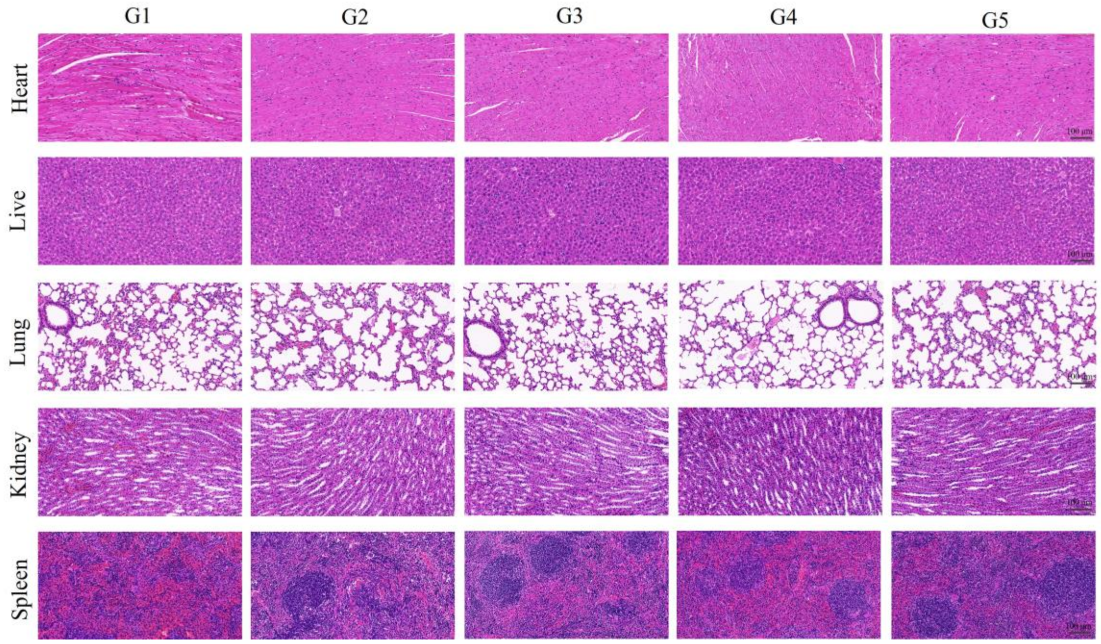

Figure S10. HE staining of heart, liver, lung, kidney, and spleen in different groups reveals non-toxicity to these important organs by different treatments. G: group.

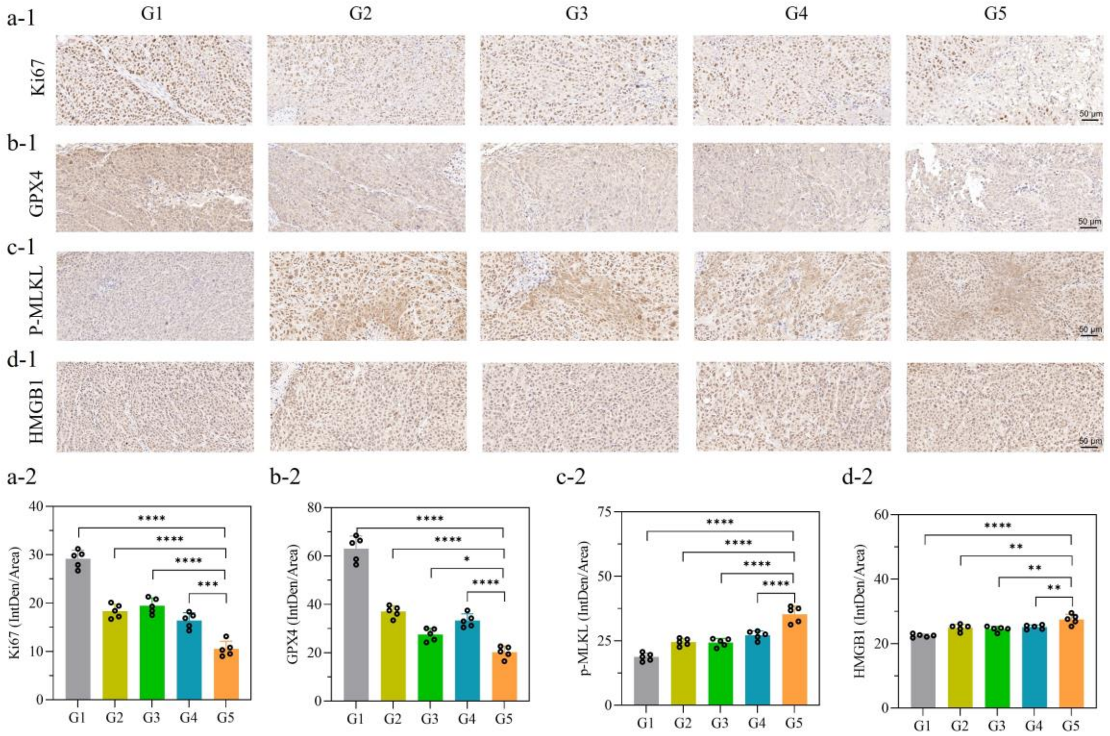

Figure S11, related to Figure 4f. IHC staining of Ki67 (a), GPX4 (b), p-MLKL (c), and HMGB1 (d) on the tumor tissues in different groups. 1: representative images; 2: quantitative analysis. n = 5. *, $P < 0.05$; **, $P < 0.01$; ***, $P < 0.001$; ****, $P < 0.0001$. G: group.

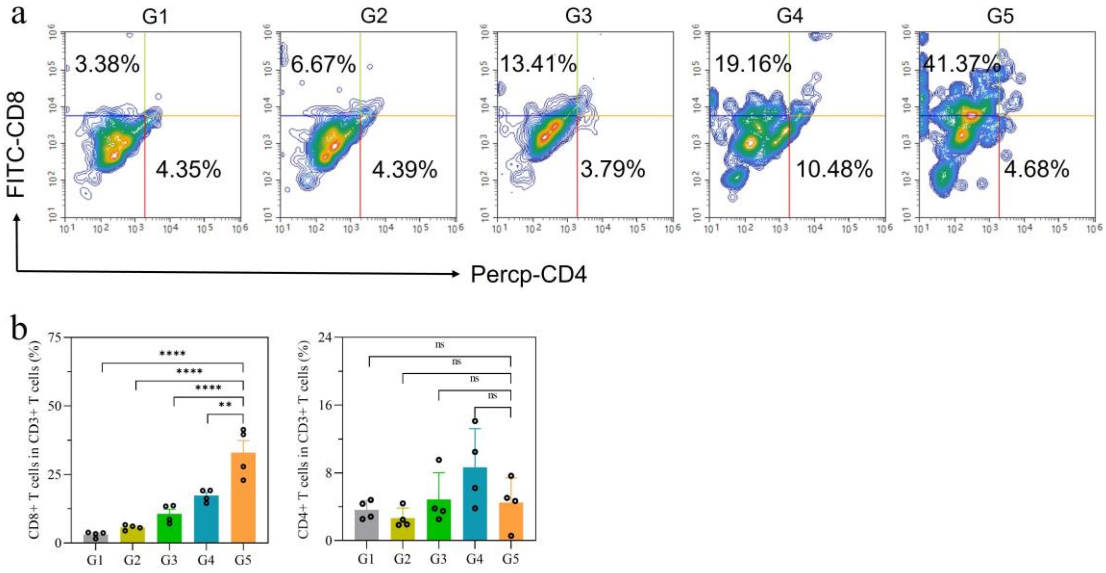

Figure S12, related to Figure 5d. Subtype analysis of T cells. (a) Measurement of $CD3^+$ T cell subtypes by CD4 and CD8 using flow cytometry. (b) Quantitative analysis on T cell subtypes indicates that $CD8^+$ T cells were significantly increased in G5. n = 4. **, $P < 0.01$; ****, $P < 0.0001$; ns, not significant. G: group.

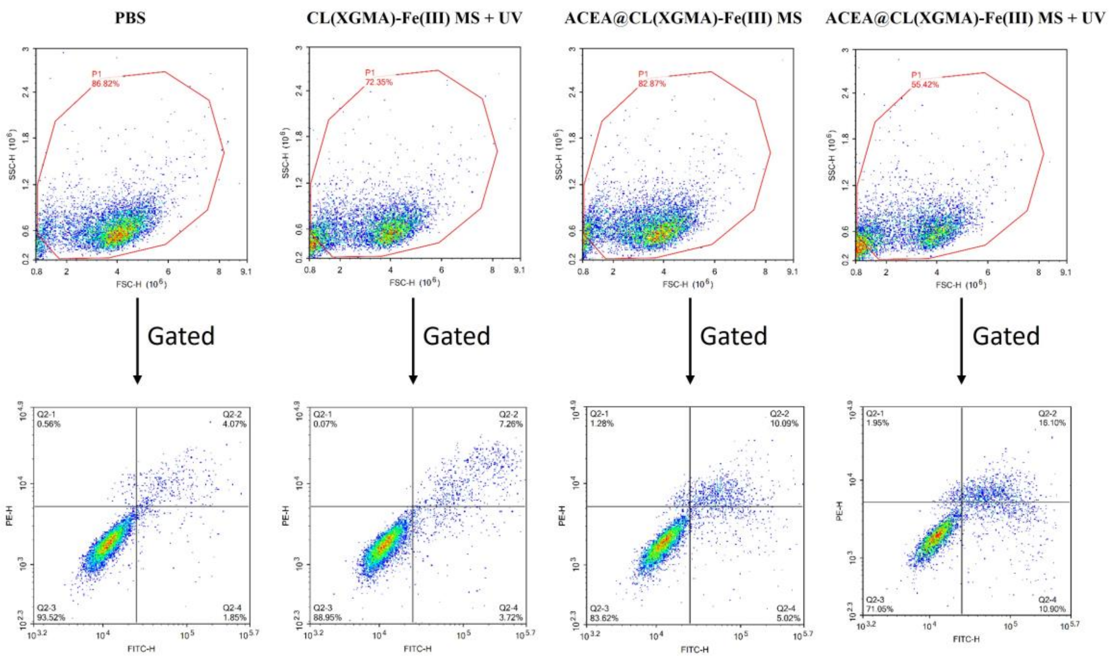

Figure S13. Gating strategy of flow cytometry to detect cell apoptosis. MS: microsphere; UV: ultraviolet.

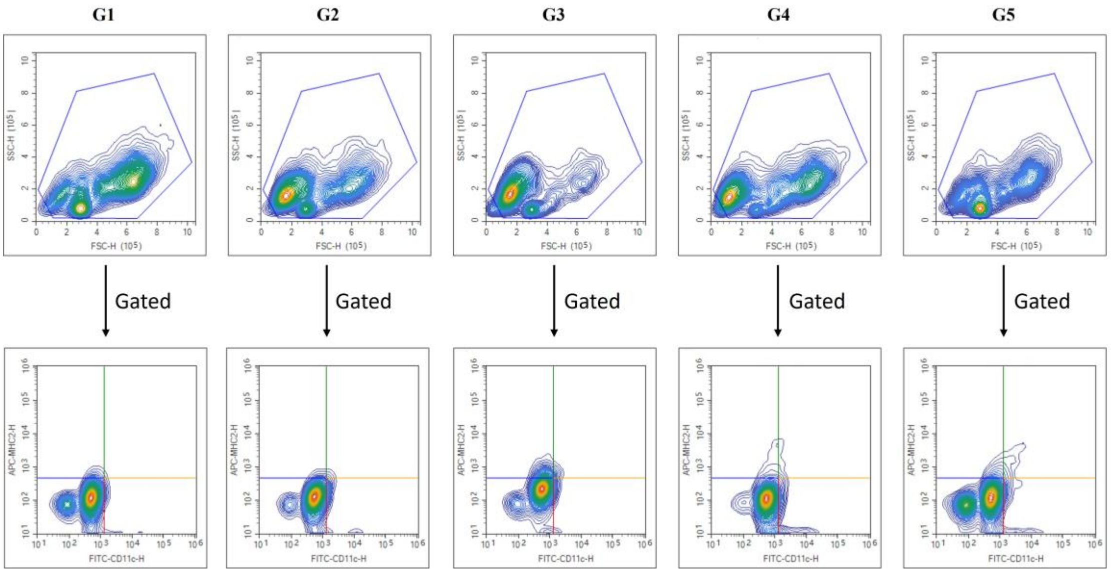

Figure S14. Gating strategy of flow cytometry to detect activated DCs (CD11c$^+$MHCII$^+$). G: group.

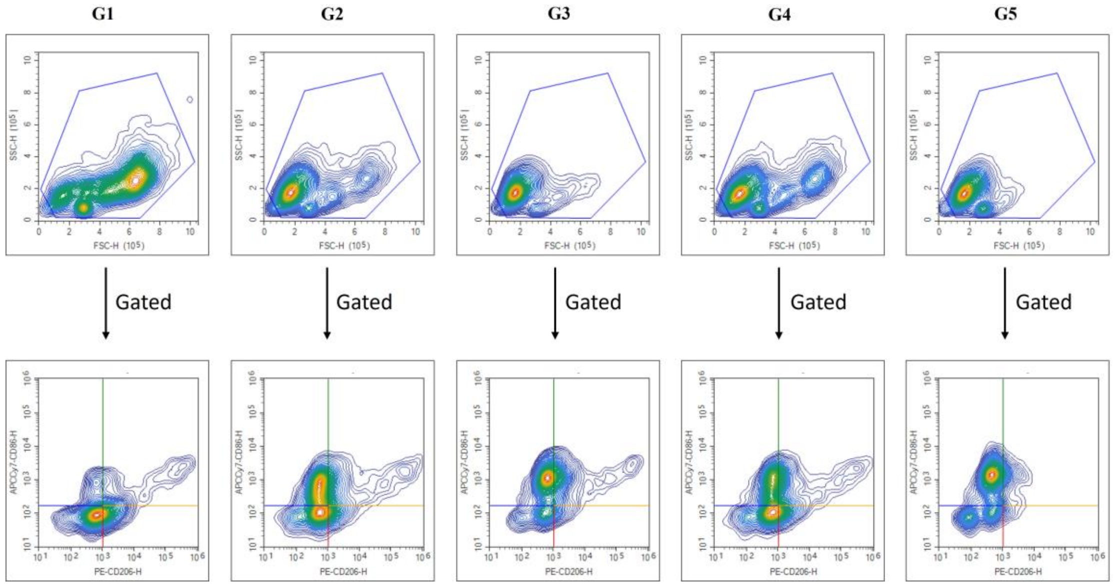

Figure S15. Gating strategy of flow cytometry to detect CD86$^+$ cells and CD206$^+$ cells. G: group.

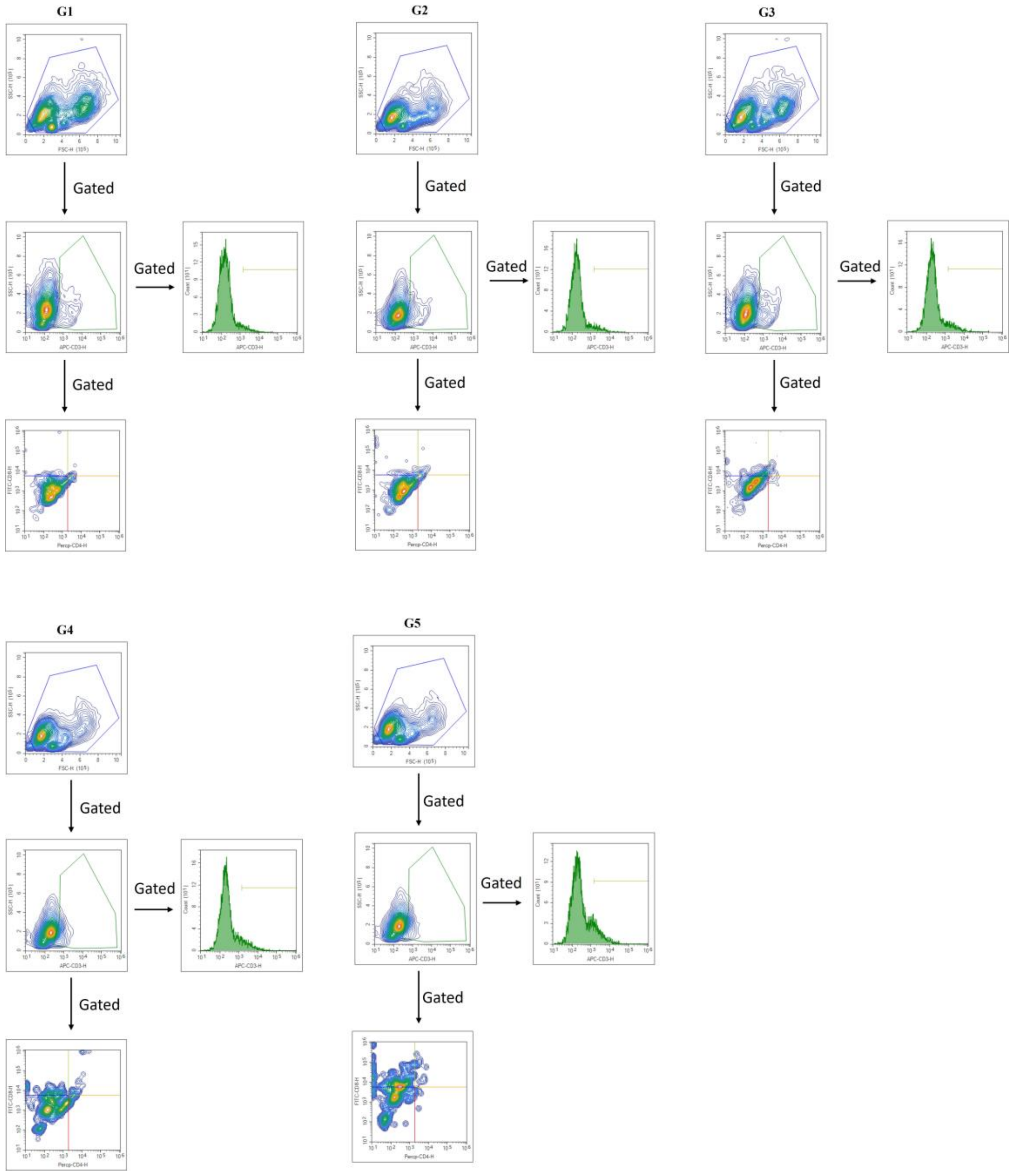

Figure S16. Gating strategy of flow cytometry to detect T cells ($CD3^+$) and the subtypes of $CD4^+$ T cells and $CD8^+$ T cells. G: group.
